# Tropism and molecular pathogenesis of canine distemper virus

**DOI:** 10.1186/s12985-019-1136-6

**Published:** 2019-03-07

**Authors:** Santiago Rendon-Marin, Renata da Fontoura Budaszewski, Cláudio Wageck Canal, Julian Ruiz-Saenz

**Affiliations:** 1 Grupo de Investigación en Ciencias Animales - GRICA, Facultad de Medicina Veterinaria y Zootecnia, Universidad Cooperativa de Colombia, Bucaramanga, Colombia; 2 Laboratório de Virologia, Faculdade de Veterinária, Universidade Federal do Rio Grande do Sul (UFRGS), Porto Alegre, RS Brazil

**Keywords:** Canine distemper virus, Canine morbillivirus, Molecular pathogenesis, Zoonosis, Tropism, Neuropathogenesis

## Abstract

**Background:**

Canine distemper virus (CDV), currently termed *Canine morbillivirus*, is an extremely contagious disease that affects dogs. It is identified as a multiple cell tropism pathogen, and its host range includes a vast array of species. As a member of *Mononegavirales*, CDV has a negative, single-stranded RNA genome, which encodes eight proteins.

**Main body:**

Regarding the molecular pathogenesis, the hemagglutinin protein (H) plays a crucial role both in the antigenic recognition and the viral interaction with SLAM and nectin-4, the host cells’ receptors. These cellular receptors have been studied widely as CDV receptors in vitro in different cellular models. The SLAM receptor is located in lymphoid cells; therefore, the infection of these cells by CDV leads to immunosuppression, the severity of which can lead to variability in the clinical disease with the potential of secondary bacterial infection, up to and including the development of neurological signs in its later stage.

**Conclusion:**

Improving the understanding of the CDV molecules implicated in the determination of infection, especially the H protein, can help to enhance the biochemical comprehension of the difference between a wide range of CDV variants, their tropism, and different steps in viral infection. The regions of interaction between the viral proteins and the identified host cell receptors have been elucidated to facilitate this understanding. Hence, this review describes the significant molecular and cellular characteristics of CDV that contribute to viral pathogenesis.

**Electronic supplementary material:**

The online version of this article (10.1186/s12985-019-1136-6) contains supplementary material, which is available to authorized users.

## Background

*Canine distemper virus* (CDV), currently known as *Canine morbillivirus*, belongs to the *Paramyxoviridae* family, genus *Morbillivirus*, and is the etiological agent of canine distemper [1]. It is considered as a highly contagious and an acutely febrile disease in dogs that has been known since 1760 [2]. It is associated with multiple cell tropism (epithelial, lymphoid and neurological), which leads to a systemic infection including respiratory, digestive, urinary, lymphatic, cutaneous, skeletal, and central nervous system (CNS) diseases [3].

The host range of CDV mainly includes species from the order *Carnivora* which belongs to the families *Canidae* (dog, dingo, fox, coyote, jackal, wolf), *Procyonidae* (raccoon, coatimundi), *Mustelidae* (weasel, ferret, fishers, mink, skunk, badger, marten, otter), *Ursidae* (giant panda), *Ailuridae* (red panda), a wide range of members of the family *Felidae* (lions, leopards, cheetahs, tigers), and in a minor extension other important families belonging to different orders such as *Artiodactyla*, *Primates*, *Rodentia*, and *Proboscidea* [2, 4]. Considering the vast array of species affected by CDV, cross-species transmission has been studied among wildlife and domesticated species in terms of the interactions among them in order to establish phylogenetic relationships [5].

**Fig. 1:**
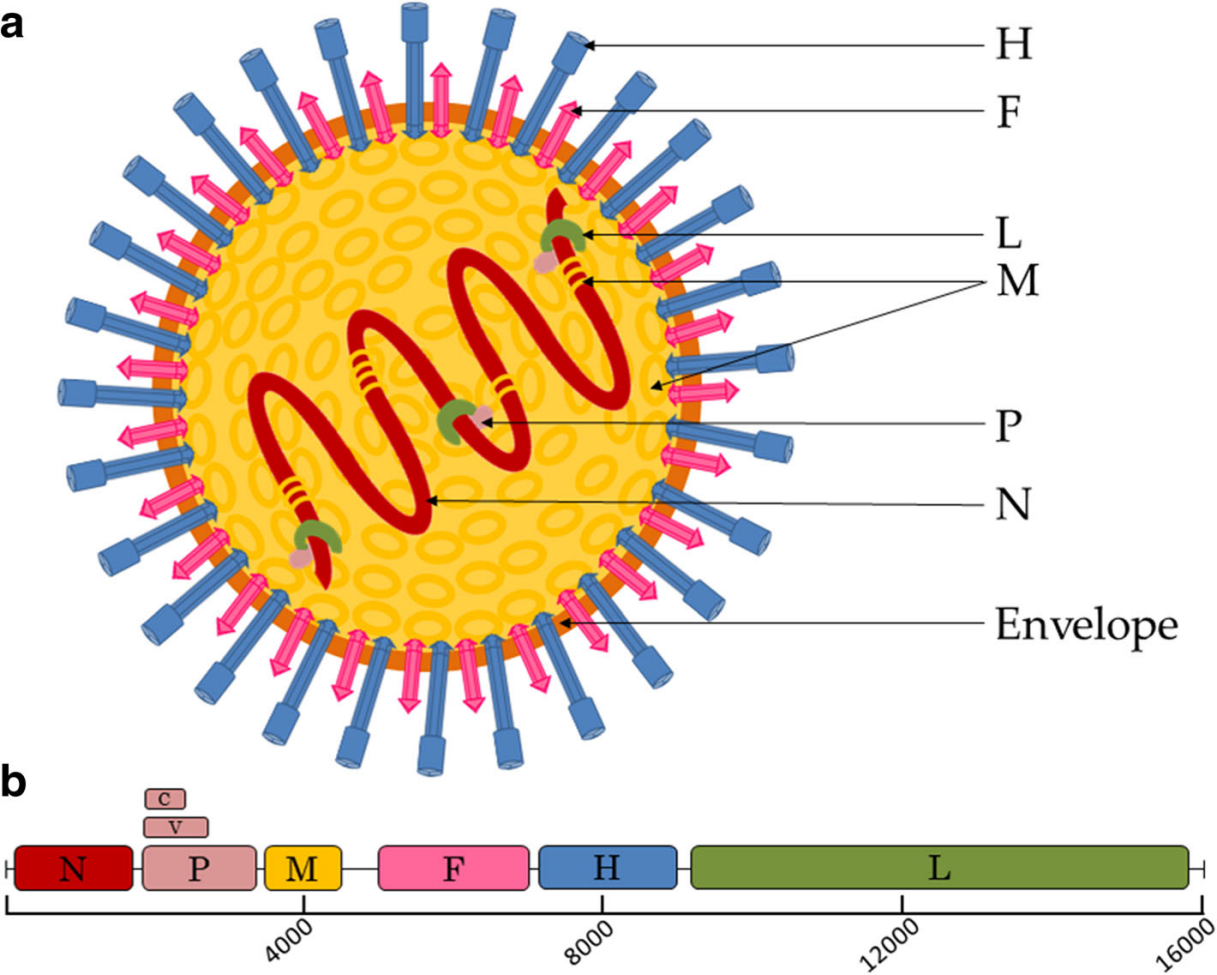
CDV virion and genome organization. **a** Schematic diagrams of CDV particle in cross-section N: nucleocapsid, P: phosphoprotein, M: matrix protein, F: fusion protein, H: hemagglutinin, L: large polymerase protein. **b** Map of genomic RNA (3′ to 5′) of CDV. Each box represents a separately encoded mRNA; multiple distinct ORFs within a single mRNA are indicated in overlapping boxes on P

The CDV particles are pleomorphic, frequently spherical, enveloped virions having a diameter of about 150 nm which include a non-segmented single negative-stranded RNA (ssRNA), similar to other members of the order *Mononegavirales* (Fig. 1a). The genome contains 15,690 nucleotides in throughout length and encodes for eight proteins [6] (Fig. 1b). The CDV genome structure includes six transcription units (N-P-M-F-H-L) organized in a linear form, which are separated by intergenic untranslated regions (UTRs) that are relatively uniform in length, with the exception of the UTR between the matrix (M) and the fusion (F) gene [7]. Those transcription units contribute to the formation of the eight proteins mentioned above. However, the P gene encodes for the C and V proteins, using an overlapping open reading frame (ORF) and RNA editing by the insertion of a non-templated G residue during mRNA synthesis, respectively [8, 9]. Both alternative gene expression strategies not only have functions that are related to transcription control and replication but also play an important role in the virus’ evasion of its host’s innate immune responses [10, 11].

All the proteins have a specific function related to the viral cycle and replication, with the nucleocapsid (N) protein encapsidating the genomic RNA, and based on the CDV gene expression, N serves as a template for the transcription and replication by the viral polymerase, denoted as L, and its cofactor, the phosphoprotein (P). The N, P, and L proteins along with the viral RNA compose the ribonucleoprotein (RNP) complex [12]. The CDV envelope involves two integral membrane proteins, the fusion (F) and the hemagglutinin (H) proteins, and finally a membrane associate protein M, which contributes by mediating the contact with the RNP, encircled by the viral envelope throughout the budding process in the host cell membrane [8]. This understanding has been arrived at mostly by using reverse genetics, which enables one to construct chimeric viruses that are to be employed in different fields such as pathogenesis studies, vaccine development, and gene therapy vectors [13].

As with other members of the *Paramyxoviridae* family, the H glycoprotein facilitates the virus binding to the host cell membrane and the F protein achieves the viral and the host membrane’s fusion, enabling the viral RNP’s entrance into the cytoplasm [12]. Two cellular receptors have been described regarding the CDV host’s cell recognition and virus entry, which include the SLAM (Signaling Lymphocyte Activation Molecule or CD150) in the peripheral blood mononuclear cells [14] and the nectin-4 (PVRL4) in the epithelial cells [15]. It has also been speculated that Nectin-4 can help the virus shed itself into the respiratory airways [16]. Based on the lack of detection of either the SLAM or the nectin-4 receptors on astrocytes, it is speculated that CDV uses an alternative receptor to invade these cells, though this potential third receptor for CDV is yet to be identified [17].

## Main text

**Fig. 2:**
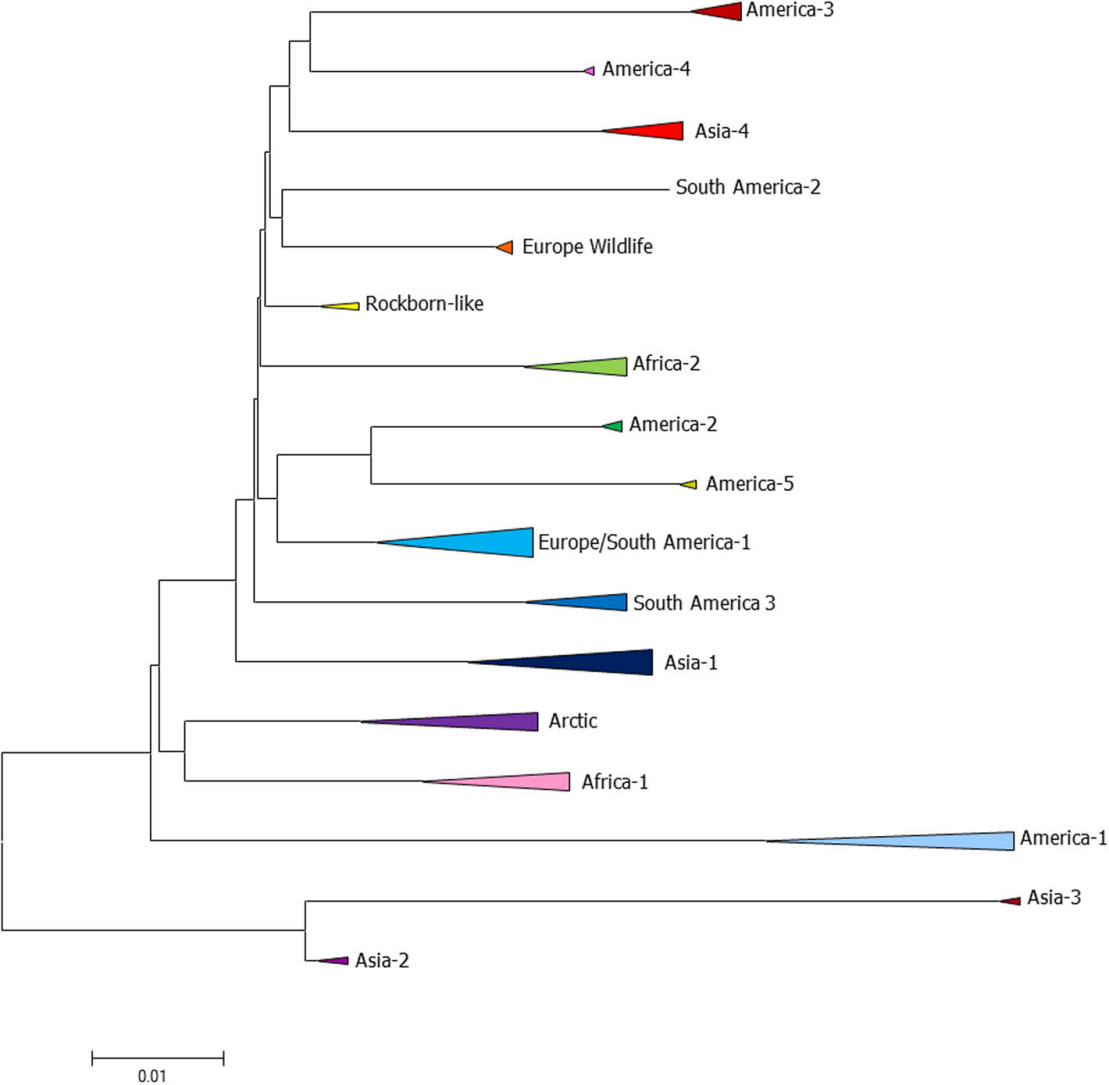
Phylogenetic tree constructed from the alignment of complete H gene sequences obtained from GenBank, which represents all current CDV-described genotypes. 67 H sequences representing all genotypes were retrieved and aligned with ClustalW using MEGA 6 software. MEGA6.0 was also used for phylogeny inference according to the Maximum Likelihood algorithm method based on the Tamura 3-parameter model. The rate variation among sites was modeled using a gamma distribution (shape parameter = 5). The robustness of the hypothesis was examined using 1000 non-parametric bootstrap analyses. GenBank accession numbers of all isolates used to construct the tree are listed in Additional file 1: Table S1.

The H protein has become the most suitable target to investigate the CDV variability and evolution. It is considered the most genetically variable gene, with up to 11% nucleotide divergence among CDV strains. This fact has enabled the conduction of CDV phylogenetic and phylodynamic studies based on genetic divergence and molecular epidemiology, respectively [12, 18]. Phylogenetic studies based on the complete sequence of the H gene from several CDV strains detected in a variety of geographic locations worldwide have been conducted to infer the genetic diversity of the CDV. The genotyping classification takes into account that within each genotype the nucleotide divergence should be less than 5% [19]. Following this criteria, to this date 17 distinct genotypes have been described: America-1 (that includes almost all commercially available vaccine strains), America-2 to 5, Arctic, Rockborn-like, Asia-1 to 4, Africa-1 and 2, European Wildlife, Europe/South America-1, South America-2 and 3 (Fig. 2) [20–30].

Concerning the CDV vaccination, an attenuated CDV vaccine was released in the 1950s and its widespread usage helped to control the CDV disease in many countries [2]. In the last few decades, however, an increase in the canine population has resulted in sporadic cases and massive outbreaks of the CDV disease, even in animals that have been vaccinated, both domesticated animals and wildlife [31, 32]. It has been suggested that antigenic differences among the vaccine strains and the circulating wild-type strains may be a causal agent [33], as the amount of CDV genotypes have increased in the recent days.

CDV has been considered a surrogate model for *Measles virus* (MeV), which is a closely related morbillivirus. Both viral agents cause a similar overall pathogenesis. However, humans and non-human primates comprise the only reservoir for MeV [8]. These facts enable one to believe that an extensive study concerning CDV pathogenesis and tropism based on the experimental data regarding MeV is necessary to elucidate the causes of occasional CDV outbreaks led by viral evolution and evasion of host innate immune response.

The equivalence among those viral models and the existence of methodologies such as reverse genetic studies has allowed the employing of recombinant virus to evaluate the effect of changes in viral genomes, particularly aspects regarding the viral life cycle and molecular pathogenesis. Although reverse genetics applied to viruses belonging to the *Mononegavirales* order have not been as efficient as expected, new technologies have been used to increase the rescue efficiency, turning it into an appropriate tool to investigate the basic aspects of the biology of viruses including CDV and MeV. This includes studies on the molecular determinants of virus entry and spreading between cells, besides the development of live attenuated vaccine vectors [34].

Initially, CDV was rescued from a full-length cDNA clone based on the Onderstepoort strain, similarly to what was being previously done for MeV and *Rinderpest virus*, through which obtaining a recombinant CDV which had no differences with the Onderstepoort strain regarding the disease progression and syncytia formation, with the exception of a genetic tag comprising two nucleotide changes that was introduced on the coding region of the L protein [35]. The introduction of the green fluorescent protein (GFP) into the cDNA clone to study the infection of the virus in a cell culture and in an animal model has been proven to be very useful in MeV studies [36, 37]. For CDV, the neurovirulent Snyder Hill strain was rescued, expressing enhanced GFP (eGFP) or red fluorescent protein (dTom), enabling a sensitive pathological assessment of the routes of virus spread in vivo; this showed how the virus rapidly circumvents the cerebrospinal fluid barriers and induces a dramatic viral meningoencephalitis [38]. A wild-type strain, 5804, that is highly pathogenic for ferrets, was also rescued, expressing GFP, retaining full virulence, and illuminating the lymphocyte-based pathways through the immune system of its infected host [39]. The roles of morbillivirus receptors SLAM and nectin-4 in transmission have also been assessed by reverse genetics. Recombinant CDVs (rCDVs) with mutations in residues of the H gene, unable to recognize one of the receptors (SLAM-blind and nectin4-blind), were generated and inoculated in ferrets, showing that both SLAM and nectin-4 receptors are required for transmission, demonstrating the importance of sequential use of both receptors in CDV pathogenesis and transmission [40]. Assessing the viral entry, intra-host dissemination and inter-host transmission, by using recombinant viruses expressing multicolor fluorescent proteins (green, red or blue) for in vivo competition and transmission, have exhibited that CDV enters the host competently when inoculated through the nose or lung and that infection of the host through conjunctival administration, although less efficient, is also possible [41]. However, reverse genetics is helpful not only to understand the molecular pathogenesis regarding the role of proteins in viral life cycle, spread, and transmission, but also for the development of vaccine vectors. Viral vectors expressing CDV glycoproteins, H alone or in combination with the F protein, have been tested as live attenuated vaccines, while one based on the canarypox vector expressing H and F proteins is commercially available. Recombinant NYVAC vaccinia virus and the ALVAC canarypox virus expressing CDV H/F have been tested and both protected against the development of symptomatic distemper [42]. Rabies virus (RABV) is also an efficient and safe platform for the generation of recombinant (rRABV) bivalent vaccines, also expressing H/F CDV proteins. An attenuated rRABV-CDVH expressing only the H protein can offer whole protection against challenge with virulent CDV in dogs [43], while a similar approach using an inactivated version generated only partial protection after the wild-type challenge of ferrets. However, the inactivated rRABV expressing both H/F proteins fully protected ferrets from lethal CDV challenges, demonstrating the critical role of immune responses directed against the F protein for the control of CDV and, likely, other morbillivirus infections [44].Conversely, CDV has also been used as a viral vector for the expression of the G glycoprotein of RABV. Animal studies demonstrated that rCDV-RVG was safe and efficient against challenges in mice and dogs [45].

### Clinical outcome

In terms of clinical characteristics, when a dog is infected with CDV, a catarrhal and a nervous manifestation, or a combination of both, and a chronic nervous manifestation can be observed. At the acute stage, viruses can be found in every secretion of the given animal [46]. This phase is followed by various clinical signs including an onset of cutaneous rash, serious nasal and ocular discharge, conjunctivitis and anorexia, followed by gastrointestinal and respiratory signs, which are often complicated by secondary bacterial infections and neurological disorders [2, 47].

The nervous signs may include myoclonus, nystagmus, ataxia, postural reaction deficits, and tetraparesis or plegia [48, 49]. However, animal recovery can be promoted by an improved immune system mostly by increasing the production of virus-specific neutralizing antibodies [50]. Albeit the fact that the virus is eliminated from different organs and peripheral blood, CDV can remain in some tissues including uvea, CNS, lymphoid organs, and footpads. Furthermore, some infected animals exhibit a retarded and diseased development and a moderate immune response with some imperceptible early clinical signs [51].

In the aftermath of the viral infection of the CNS, some disturbances can be perceived. Generally, dogs with CNS pathologies do not survive. However, some may recover and exhibit lifelong neurological symptoms [3]. Demyelinating leukoencephalitis (DL) is also commonly induced by CDV in the latest stages of the disease, and in terms of immunopathological processes, glial responses and early axonal degeneration, DL by CDV shares some characteristics with other diseases that cause demyelination, such as multiple sclerosis either in human or animal models [52]. It has been previously studied that the chronic phase of CDV infection generates DL [3]. The decrease in viral titers, the alteration of maturation, and the plasticity of astrocytes, primary axonopathy, and a probable role of Schwann cell-mediated regeneration are crucial events in DL [3]. Virus persistence in the CNS can be observed with some CDV strains, for instance the A75/17 strain, when the virus is capable of spreading to some areas of the brain without eliciting an inflammatory response. This strain is also known to infect cell lines very inefficiently, with limited syncytia formation [53].

### CDV proteins and their role in pathogenesis

The CDV genome encodes eight proteins within six transcription units. CDV proteins have a specific activity regarding virus replication and in the infection cycle. The N protein not only provides the basis of the helical structure of the RNP, but it is also a requisite in some replicative viral processes [54]. Among its various functions, the N protein protects the genome from degradation, avoids the formation of dsRNA between viral RNAs of opposite polarity, and packs the RNA into the RNP. Moreover, as a consequence of its dynamic interaction with RNA and the L protein, it controls the L access to the RNA within the template RNP and nucleates the assembly of progeny RNPs. Consequently, the N protein due to its interaction with the genomic RNA [36] controls both the replication process and the transcription process [49]. On the other hand, the L protein shows the polymerase activity and is carried by the virus particle. The P protein works as its cofactor, which has two fundamental functions. The first is to recognize the RNP as the polymerase template and the other is the stabilization of the nascent N protein [54].

The H and F proteins’ main functions are to mediate the recognition, attachment, and fusion processes of the CDV to the host cell. The attachment protein H, which lacks the neuraminidase action observed in other viruses, attaches to receptors present on the plasma membranes of host cells, such as SLAM, nectin-4, and other, in glial cells [55]. Moreover, the M protein is essential in the assembly and budding of CDV particles, and acts as an intermediate between the RNP and the glycoprotein surfaces by enabling the interaction of M with the C- and N-terminal of N and the cytoplasmic tails of H and F proteins [56]. V and C proteins are non-essential with respect to the virus replication process but critical for preventing the host immune responses. Therefore, cooperative actions between them can be critical to efficiently evade the host’s immune responses and cause diseases in vivo [57].

Hence, besides the CDV protein’s functions, it is important to mention that all molecular interaction depends on the nature of the molecules and the amino acidic sequences, which together define all protein functions. These factors influence not only the host cell responses but also the CDV infection cycle, and clearness in this molecular process is essential to the understanding of CDV cell tropism and pathogenesis. In the following paragraphs, we will try to address the role of each of the CDV proteins in virus replication, life cycle, and pathogenesis in further detail.

### Infection cycle

Based on the presence of F and H glycoproteins, CDV can successfully overcome the plasma membrane, which is considered as the host’s first cell defense barrier, through a hetero-oligomeric complex composed of a tetrameric H and a trimeric F. The H glycoprotein has been widely implicated in the interaction with specific host cell receptor, by recognition of specific amino acids, followed by multiple conformational changes in H and F. It has been recently shown that there exists a central pocket in the globular head domain of F that regulates the stability of the metastable, pre-fusion conformational state of the F trimer [58]. This interaction is mediated by two hydrophobic residues located in the Ig-like domain of the F globular head domain, which contributes to the interaction between the receptor and the membrane-proximal domain of the H stalk [46]. In addition, it has been reported that the intensity of the F-triggering stimulus obtained by the H tetramers is influenced by the origin of the H protein and the molecular nature of the contacted receptor [58]. This is due to the fact that critical residues are located at the front H-binding site that has been implicated in the step of inducing such fusion machinery [59]. Such structural rearrangements in the protein complex facilitates the virus’ attachment on the cell membrane surface, the formation of fusion pores, and thereafter the introduction of RNP complex into the host cell cytoplasm. Membrane fusion is also required for cell-to-cell spreading of the virus, which results in a multinucleated cell formation known as syncytia, which is a remarkable cytopathogenic characteristic of the morbillivirus [60].

**Fig. 3:**
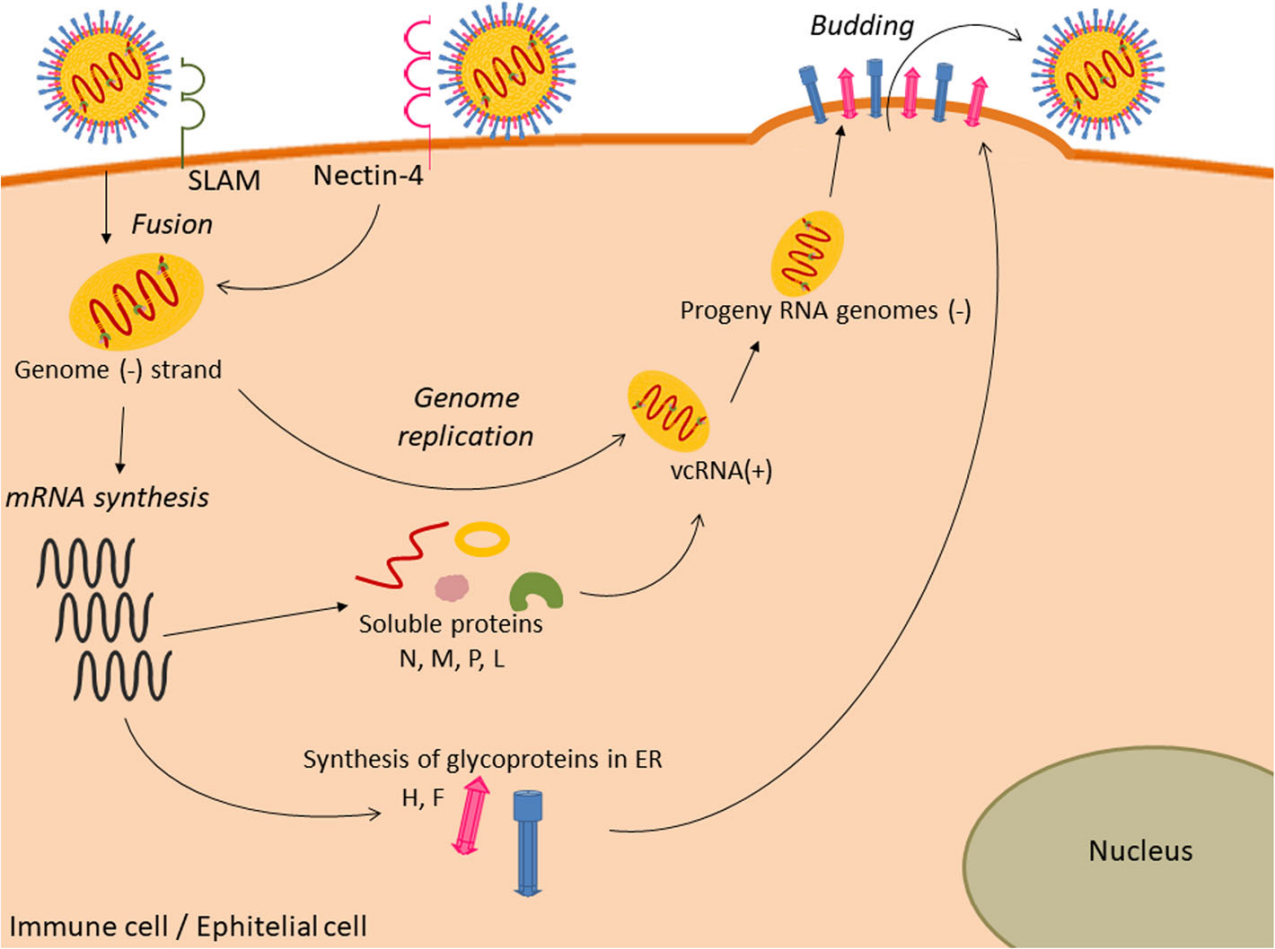
Replication of the CDV cycle. Virus particle recognition by host cell receptors (CD150 or nectin-4), RNP release into de cytoplasm, replication, transcription process, and virus particle budding are illustrated

All the CDV replication and transcription strategies are similar to that of the other members of the *Mononegavirales* order, as shown in Fig. 3 [61]. The polymerase complex is formed by two different proteins: the subunit L, liable to the enzymatic process with its domains being involved in RNA synthesis, capping, and cap methylation, and the phosphoprotein P, an essential cofactor related to the functioning of L [62].

An important characteristic of the genome template in the CDV is associated with the nucleoprotein N in terms of constituting a helical nucleocapsid and forming the N-RNA structure [63]. While the polymerase proceeds, it recognizes the beginning and ending gene signals and generates six sub-genomic mRNA. Therefore, at a beginning gene signal, the polymerase starts the mRNA synthesis and at the ending gene signal, it releases the synthetized RNA. Afterwards, the polymerase surveys the intergenic regions to locate the next beginning gene signal. This process is done with each gene and there is evidence of a methyl cap addition and a poly A, both of which are essential for the polymerase to change into the elongated mode [63, 64]. Interestingly, the P ORF has also an RNA-edited form, where the RNA transcriptase hesitates on the RNA template at an RNA editing motif, leading to the addition of a pseudo-template guanine. As a result, the V protein has the same amino-terminal domain as a P protein, but has a different carboxy-terminal domain. In opposition to the V protein, C mRNA transcription is initiated at an alternative start codon [64].

As mentioned before, the morbillivirus genome is composed of six transcriptional units that are separated by untranslated regions (UTRs) which are relatively uniform in length (approximately 100 to 200 nucleotides), with the exception of the UTR between the M and the F genes, which is at least three times longer and highly variable [65]. It has been documented that the F 5′ UTR of CDV is essential in translating an unusually long F signal peptide (FSP) [66]. This signal is quite different from a classical signal sequence as this region has regulatory functions in vitro as in vivo (in ferret models of disease), indicating that the region between CDV M and F genes modulate virulence by controlling the F protein’s expression [67]. On the other hand, a short putative ORF has been identified within the wild-type CDV-M 3′ UTR [68]. The proximal part of the M 3′ UTR modulates the initiation of viral genome replication and is involved in the disease’s prolonged extension in ferrets, indicating that both specific sequence elements as well as the general length are required to maintain a wild type virulence [65, 69].

Similar to the MeV replication cycle, it is crucial to assemble the M protein, which plays an important role in the assembly and budding of virions, considered as an intermediary between the RNP and the surface glycoproteins and orchestrating the viral particle assembly process [56, 70]. The RNP and the glycoproteins, in specific regions of the plasma membrane of the host-infected cells, form complete infectious CDV particles as the result of a coordinated interaction between viral and cellular factors [71]. As proven, the C-terminal of the N protein is fundamental in its interaction with the M protein; mutations or deletions within it inhibit the transport of the RNP complex to the plasma membrane through infection, demonstrating the importance of the M protein in the integration of the RNP complex into the CDV particles [71]. For the MeV, it has been demonstrated that mutations in the M protein or deletions of the whole protein diminishes virus assembly and influences pathogenesis [70]. Based on these facts, the M protein leads to CDV assembly and budding in spite of the deficiency of other proteins, considering that the paramyxovirus M protein is sufficient to shape virus-like particles [71]. CDV budding is believed to be independent of the cellular Endosomal Sorting Complex Required for Transport (ESCRT) machinery for the host cell egress, which is particularly conducted by the CDV M protein [72].

Lastly, it is believed that the F and the H proteins are assembled in intracellular milieu. The M protein attaches itself to the RNP complex in the cytoplasm and carries it to the plasma membrane, where the F and the H proteins are convened with the budding virus particle. This may be related to the observation that the CDV envelope proteins, H and F, are partitioned into cellular detergent-resistant membranes (DMRs), which may form the structural basis for membrane rafts. Consequently, the role of lipid rafts in the virus assembly as well as the release is suggested, as there is a necessity of the virus-enveloped cholesterol since the depletion of cholesterol in the cell membrane harboring the CDV envelope proteins resulted in the decrease in syncytium formation. Hence, both the incorporation of the envelope proteins into DRMs and their interaction with cholesterol may be necessary for the virus’s entry and release [73]. Furthermore, it has been shown that the first 10 residues from the CDV H cytoplasmic domain strongly influence its incorporation into virus-like particles that are formed by the CDV matrix (M) protein. In addition, this domain is required to ensure the correct translocation of nascent proteins into the endoplasmic reticulum to suffer post-translational modifications [74].

### Tropism and pathogenesis

CDV is considered a multi-cell pathogen that has the ability to infect three different types of host cells including epithelial, lymphoid, and neurological cells. Infections may occur not only by the inhalation of aerosol droplets or airborne virus particles but also as a result of direct contact with bodily fluids or through fomites [41]. Contemplated, as a systemic infection and affecting a vast array of organs and tissues, there are some CDV host cell receptors that have been widely studied, such as the SLAM, which is expressed on activated T- and B- lymphocytes, and dendritic cells (DCs) and macrophages. These behave like the regular entry receptors for morbilliviruses. Other extensively studied receptors include nectin-4, which is recognized as an epithelial cell receptor and currently considered to function as a host exit receptor [75, 76].

**Fig. 4:**
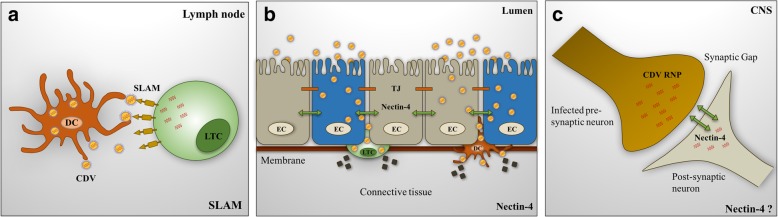
Principle routes of CDV infection and transmission in hosts. **a** Infected DCs and alveolar macrophages progress to the local draining lymph node, where they interact with and infect T-cells and B-cells through CD150 that is also expressed on their cell surface. These infected cells further spread to secondary lymphoid organs, causing a secondary viremia. **b** At the final stages of infection, shedding of infected lymphocytes to distal site of the respiratory tract. These infected lymphocytes interact with the epithelial cell receptor nectin-4, located in the adherent junctions on the basolateral surface of the epithelial cell. Infection in the airway epithelium results in the virus assembly and the release of virions into the airway lumen of the infected lung. **c** CDV can infect the CNS in some instances and it has been suggested that the receptor nectin-4 has an important role in this infection

Based on MeV, in the first stages of infection within the host, resident DCs and alveolar macrophages in the respiratory tract are infected along with other cells which express CD150 in the alveolar lumen [64]. Similar to the MeV, CDV H protein attaches itself to the cell via the CD150 cell receptor [77]. It is believed that there is a translocation of an intracellular pool of CD150 into the cell membrane surface. Infected cells carry the virus to the draining lymph node where then the resident activated T-cells and B-cells are infected through the CD150 receptor, resulting in virus amplification and the initiation of primary viremia (Fig. 4a) [78]. The virus gets disseminated to secondary lymphoid organs, including the spleen, the thymus, the tonsils [39] and subsequently a systemic spread through the entire immune system. The decline in the amount of white blood cells (WBCs) or leukocytes is remarkable (leukopenia) besides an inhibition of non-specific lymphocytes, which continue increasing while the CDV gets disseminated throughout the immune system [79]. Due to detectable infection levels in peripheral blood mononuclear cells (PBMCs) are not significant at this point of infection; the migration of this kind of immune cells to the infection site must be an essential factor that contributes to leukopenia as well as virus-induced cell death. This immune suppression causes some opportunistic and secondary infections to arise, contributing to the morbillivirus’ morbidity and mortality [80].

Dissemination of the virus to distal sites including liver, skin, gastrointestinal tract, genitals, and respiratory mucosal surfaces results in the virus’ spreading and subsequent transmission to uninfected individuals (Fig. 4b) [78]. Throughout the respiratory tract, CDV infection is thought to occur via the basolateral side of the lumen epithelium via the migration of CDV-infected T, B, and DCs from the circulation [39]. At this point, nectin-4 is located within the adherent junctions and set to interact with the viral particles that have been carried onto the surface of these infected lymphocytes, thus enabling the virus’ entrance to the epithelium. CDV exits from the epithelium via its apical surface. In the absence of no nectin-4, CDV remains lymphotropic and produces primary and secondary viremia. Thus, the epithelial receptor-joined virus is rendered incapable of spreading from the respiratory route, suggesting that nectin-4 plays an essential role in virus egress late in the infection rather than during its initial stages [16, 39, 81].

A previous study reported that CDV infection through an epithelial receptor is required to have the clinical disease but not necessary for immunosuppression, deriving from the fact that after animals where inoculated with epithelial receptor-blind CDV strains (which lack the epithelial cell receptor recognition domain) they showed no clinical signs. However, there was a rapid and efficient spreading of immune cells, producing the same levels of leukopenia and inhibiting lymphocyte proliferation activity which are signals of morbillivirus immunosuppression [82]. Additionally, it has been confirmed through in vivo experimentation in ferrets that transmission was not evident in most animals infected with the SLAM- or nectin-4-blind CDV strains obtained by reverse genetics systems, although all animals infected with the nectin-4-blind virus developed continuous viremia, remarking the importance of epithelial cell infection and sequential CDV H protein interactions—at the beginning with SLAM and then with nectin-4 receptors—regarding transmission to naive hosts [40]. This fact also highlights the importance of in vivo selection pressure on the CDV H protein interactions with SLAM receptors.

Respiratory and gastrointestinal clinical pathologies are the most common signs by the end of 6 to 10 post-infection days along with rashes (a typical symptom of CDV) in the form of erythematous patches whose diameter ranges between 3 and 8 mm. The neck and the face are the first body parts that are affected. An increasing number of patches appear around the mouth while the infection progresses [64, 79]. Furthermore, the skin becomes another target of the virus. Similar to other morbilliviruses, CDV can infect the epidermal cells of a wide range of species. Footpad keratinocytes are commonly infected, since the demonstration of viral antigen in the biopsies of the footpad serve as a diagnosis for CDV [83].

### Neuropathogenesis

CDV has been also studied as a neurotropic agent; a vast number of strains are responsible for polioencephalitis and, predominantly, several of them generate DL. As is believed, the CDV must reach the brain in different ways, and some infection routes have been suggested thus far. One of the most crucial routes of neuro-invasion extend via infected PMBCs that are transported through the blood brain barrier; afterwards, there is a virus release that results in the infection of resident epithelial and endothelial cells [3]. In vivo, using a mouse model of infection, it has been proven that the CDV infection progresses from circulating cerebrospinal fluid into the CNS through a sequential route by infecting the neuro-ependymal cells lining the ventricular wall and the neurons of the hippocampus and the cortex that lie adjacent to the ventricle area. It then causes an extensive infection of the brain’s surface, followed by the parenchyma and the cortex [84].

However, in ferrets, it has been proven that the CDV can enter the brain via neurons located in the olfactory mucosa, invading the olfactory nerve filaments, the olfactory glomeruli, and the deeper CNS structures. As mentioned before, the cellular receptors which contribute to neuropathology include nectin-4 (Fig. 4c) [78] and a third in astrocytes [85], due to the SLAM receptor being under-expressed in the CNS [3, 86]. Furthermore, it has been reported that viral persistence and neurological disease is related to the CDV in viral cell-to-cell spread in astrocytes, allowing the virus to avoid the immune system detection. This CDV infection is based on membrane fusion between the infected and the target astrocytes that heralds a free passage of viral nucleocapsid [87]. Additionally, it has been demonstrated that the functional hetero-oligomeric viral H/F complex, and thus presumably membrane fusion, are required to enable the spread of CDV in primary astrocytic cultures [88]. Consequently, the CNS, the astrocytes, the microglia, the oligodendrocytes, the neurons, the ependymal cells, the choroid plexus cells and, as demonstrated by most rigorous studies on CDV, a family of growth-promoting glia including some specialized macroglia with a Schwann cell-like structure can be infected, enabling the development of neurological CDV infection [47].

It is relevant to mention that as an implication of the neuropathology of CDV, the early stages of DL are a consequence of a direct virus-mediated damage and the invasion of CD8+ cytotoxic T cells, which are associated with an up-regulation of pro-inflammatory cytokines such as interleukin (IL)-6, IL-8, tumor necrosis factor (TNF)-α, and IL-12, and a lack of responses from immunomodulatory cytokines such as IL-10 and the transforming growth factor (TGF)-β. CD4 + -mediated delayed type hypersensitivity and cytotoxic CD8+ T cells contribute to myelin loss in the chronic phase. Furthermore, an up-regulation of interferon-γ and IL-1 must occur in advanced lesions [47]. Once a dog has overcome the immunosuppression induced by the CDV infection, immune-mediated demyelination can be noticed, as MeV does in humans, along with some aspects of the inclusion of body encephalitis. Consequently, old dog encephalitis occurs a long time after one’s recovery from a CDV infection and shares clinical and pathological features with the MeV subacute sclerosing panencephalitis [64].

### Host-virus interaction with and evasion of the immune system

The function of V and C proteins in CDV pathogenesis still lies within the interests of researchers. Studies have shown their incidence towards CDV pathogenesis and the counteraction of the host interferon (INF) responses. The association of an innate immunity and virus-mediated immunosuppression impacts the infection development [64]. The immune response is initially activated by diagnosing the pathogen-associated molecular patterns (PAMPs) through pattern-recognition receptors (PRR) including Toll-like receptors, melanoma differentiation-associated factor 5 (MDA-5), retinoic-acid inducible gene (RIG)-I-like receptors, and nucleotide-binding oligomerization domain-like receptors (NLRs). The definition of a viral infection by PRR guides us to interferon regulatory factors (IRF)-3, IRF7, and nuclear factor kappa-light-chain-enhancer of activated B cells (NF-κB). As a result, not only are the production of type I IFN observed but the inflammatory cytokines [64] are also identified.

In general, for the *Paramyxoviridae* family, V protein interacts with RIG-I-like receptors, MDA-5, and LGP2, suggesting a positive regulator of RIG-I- and MDA-5-mediated antiviral responses meant to suppress IFN induction. Moreover, the virus escapes detection by MDA-5 by interacting with other cell factors [64]. By using in vivo models, it has been reported that the V protein of CDV is critical in the inhibition of IFN-α/β induction in PBMCs and of other important cytokines such as the tumor necrosis factor alpha (TNF-α), gamma IFN, IL-6, and IL-4 that controls cellular and humoral immune activation [11]. Detailed molecular analysis by using the neuropathogenic CDV A75/17 strain has demonstrated that the V protein specifically ablates the nuclear import of STAT1 and STAT2 without affecting their activated phosphorylation states [89]. Furthermore, the inhibition of IFN-α/β-dependent signaling is correlated with the capacity of the V protein to efficiently interact with both STAT molecules with both the N-terminal and the C-terminal regions of V, playing a synergistic role in the IFN evasion [89]. Additionally, a domain of V protein, which is shared with the P protein due to its alternative gene expression, can obstruct Type I and Type II IFN responses. This action is attributed to amino acids 110 and 130 with the tyrosine 110, which is an essential an amino acid in terms of binding to the signal transducer and the activator of the transcription 1 (STAT1) molecule. As reported, inhibition of the V protein results in a 70% loss of Type I IFN inhibitory action, since this region directly inhibits IFN-β synthesis through its interaction with MDA-5 [90].

Through systematic mutagenesis, it has been revealed that both aspartic acid 248 and phenylalanine 246 are essential for the inhibition of STAT2 nuclear translocation. Comparatively, arginine 235 is necessary for MDA-5 interference in paramyxovirus. Thus, the importance of the V protein regarding morbillivirus virulence and particularly in relation to MeV and CDV has been demonstrated, due to its relationship with the host cell factors. This also indicates that the V protein sequence may be crucial in the molecular interaction and modulation of the host’s immune responses [90].

### H protein structure as the key molecular factor in CDV tropism

As an attachment protein, the H protein is a monomer belonging to the transmembrane glycoprotein type II, consisting of a small N-terminal cytoplasmic tail, a transmembrane domain, and a large C-terminal ectodomain. This ectodomain is confirmed as a stalk and a six-blade (B1–B6) β-propeller fold lying near a central cavity. Each blade holds four-stranded anti-parallel β-sheets (S1–S4) [12, 91]. It has been postulated that the H protein, after binding to specific receptors on target cells, induces an oligomeric conformational change on the stalk domain, which in turn may translate into an F activation. In addition, the ectodomain stalk supports the membrane-distal cuboidal head region [58]. Many studies have evidenced that the paramyxovirus attachment protein stalk domain physically interacts with the F trimers and forms short-range contact with the large globular head domain of the trimeric F, which causes an overlapping H-F association model in which the H heads are located above the F heads [92].

Additionally, due to H protein being considered as the most variable gene in CDV strains, analyzing the incidence of amino acidic variability in initial interactions, virulence, host range, immune system responses, and neutralization of the epitopes of CDV is completely relevant [18]. Several comparative studies have demonstrated that this heterogeneity in the H proteins of diverse strains is associated with higher genetic-antigenetic variations compared other CDV genes; consequently, neutralization-related sites are affected and then one observes the disturbance of important epitopes. This genetic diversity of the H CDV gene disarranges the antigenicity of the arising CDV strains (this holds for CDV strains that are used as current vaccines), which was demonstrated by the CDV stains which were isolated in Italy [93].

Even though the SLAM receptor has been broadly studied as the CDV receptor in the determined immune cells and correlates with the immunosuppression associated with the CDV-mediated cytolytic infection of the lymphoid tissue, there is evidence behind the statement that the other types of receptors must facilitate CDV entrance to nectin-4, as the epithelial cell receptor contributes to CDV multi-tropism. Similar to MeV, 11 residues of the CDV H protein have been identified by a site-directed mutational analysis of this protein, which regulates and mediates plasma membrane recognition and posterior fusion in epithelial keratinocytes [94]. Furthermore, the SLAM receptor binds itself to the CDV H protein at specific regions which comprise 500 to 550 amino acids [94].

**Fig. 5:**
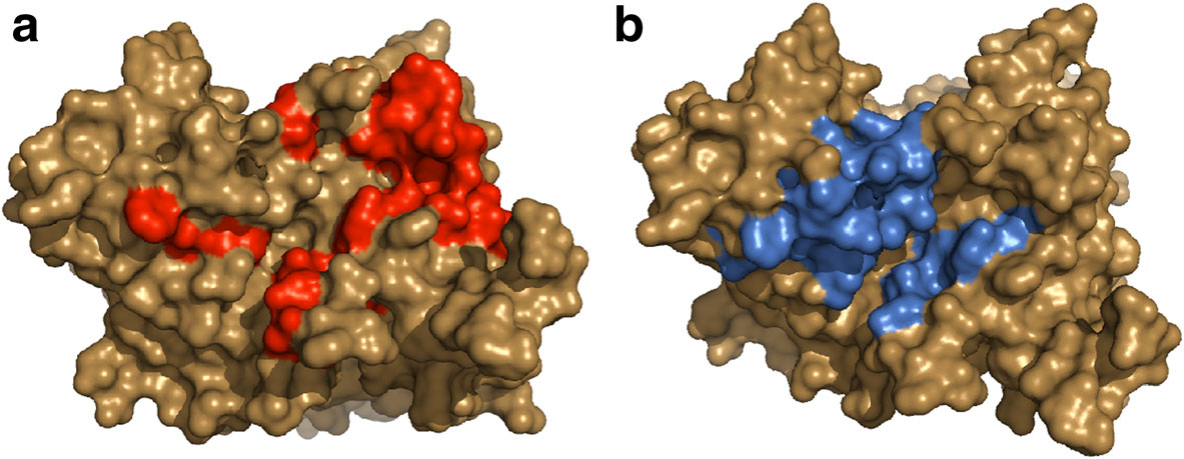
H protein from CDV reference strain, based on MeV crystal structure. Surface representation of the CDV H structure accomplished through homology modelling based on MeV H (PDB: 2RKC), using the software PyMOL, Molecular Graphics System, Version 2.0 Schrödinger, LLC. **a** In red, residues that potentially interact with mononuclear cell receptors (SLAM); **b** In blue, positions of CDV H protein that interact with the epithelial cell receptor (nectin-4). The interaction positions of CDV H protein are presented based on the interaction sites of the MeV H protein

Altogether, there are several mechanisms by which to obtain an understanding of the molecular interactions between the H protein and the host cell as the mediator of the CDV entry. Computational tools and directed mutagenesis of the H protein are some of the useful strategies to study these types of interactions. Regarding the computational biology, a crystallized CDV H structure which enables a structural study has unfortunately been unavailable till date [94]. Consequently, we have modelled a CDV H protein from a reference strain (GenBank code: AAG15490.1). Figure 5 exhibits the CDV model constructed through homology modelling with the help of Modeller, based on the MeV H protein structure (PDB code: 2RKC).

Structural studies on the H protein have confirmed the existence of interaction sites with cellular receptors. The SLAM receptor interacts with such a region that has multiple contacting sites [94], as shown in Fig. 5a [95]. Similarly, the nectin-4 receptor has established some interaction sites with the CDV H protein (Fig. 5b) [95]. These interaction sites are reported based on MeV [95, 96]. Consequently, there is structural and functional evidence that an H surface behaves as a multiple-receptor binding domain and gives an idea about high selectivity, suggesting that there might be differences between the H protein and the receptor interface, which is essential in leading the CDV entry into and infection of specific cells [94].

### CDV as a potential cross-species agent

To date, there has been no evidence of a human infection by CDV. However, as reported, it can be isolated from human cancer cell lines such as those that are observed in breast, lung, and prostate cancer. As is already known, CDV employs dog SLAM receptors efficiently, but fails to do so with human SLAM. Therefore, in contrast to this the human nectin-4 present in cancer cell lines, as mentioned before, also operates as efficiently as CDV receptors [57]. As an explanation behind this phenomenon, there is a small species-related variation in the nectin-4 sequences between humans, mice, and dogs to such an extent that mice’s nectin-4 can function as a receptor for MeV while human nectin-4 functions as a receptor for CDV. However, the mice SLAM cannot function as a receptor for either MeV or CDV, and the attachment appears to be dictated by the amino acid sequence in the V loop of this protein [97].

The V loop of nectin-4 is also involved in the process of virus attachment. Yet, there are just three amino acids that differ in the V domain of the dog homologue and six different amino acids in the V domain of a mouse, as compared to the human protein sequence [75]. In 2013, Otsuki et al. demonstrated that the Ac961 CDV strain replicates in human epithelial NCI-H358 cells, expressing nectin-4, and adapts to them. Surprisingly, no amino acid change in the H protein was required for adaptation. Therefore, the ability to use human nectin-4 is an intrinsic phenotype feature of wild-type CDV strains [98]. In 2006, the CYN07-dV CDV strain was isolated in vitro in the Vero cells that expressed the dog SLAM receptor. After phylogenetic analysis, this strain was found to be similar to the one observed during a CDV outbreak in China. However, YN07-dV uses the Macaca SLAM and Macaca nectin-4 receptors as efficiently as the dog SLAM and dog nectin4, respectively [99].

In 2014, De Vries et al. through reverse genetics generated a recombinant CDV and studied its virulence and tropism by expressing an EGFP in naïve and MeV-vaccinated *Cynomolgus macaques*, finding that in naive animals CDV produced viremia and fever by infecting lymphocytes and dendritic cells that expressed SLAM receptor [100]. These facts demonstrated that CDV could infect nonhuman primates; however, partial protection was distinguished in MeV-vaccinated macaques, as demonstrated by a controlled virus replication. Moreover, neither CDV infection nor MeV vaccination induced noticeable cross-reactive neutralizing antibodies. MeV-specific neutralizing antibody levels in MeV-vaccinated macaques were increased by CDV infection, which suggests that cross-reactive epitopes do exist [100].

In other studies, it was found that CDV isolated from monkeys (Monkey-BJ01-DV) replicates itself efficiently in Vero cells expressing the SLAM receptors and originating from dogs and monkeys. However, it does not replicate itself in cells of human origin express the SLAM receptors. In this regard, the essential cause can be the substitutions in the isolated H protein and the CDV H protein. Moreover, while the amino acid sequence identity of the dog SLAM and the monkey SLAM is only 63.6%; the Monkey-BJ01-DV strain is able to replicate itself on the Vero cells expressing the SLAM receptors and originating from dogs as efficiently as the Vero cells which originate from monkey SLAM [101], indicating a potential cross-species event.

Even though the CDV can infect non-human primates, the human SLAM’s incompatibility with the CDV attachment protein, as a consequence of its sequential difference from the dog SLAM receptor, can result in the absence of the infection in humans based on the infection cycle, since it is believed that cells expressing SLAM receptors are infected at the beginning. Furthermore, a cross-reactive immunity between MeV and CDV might be protecting humans against CDV infection [57, 88]. It has been proven that MeV and attenuated CDV have induced incomplete immune responses to canine distemper disease [102]. Other studies have compared both MeV and CDV vaccines for the prevention of canine distemper in young dogs, which were also challenged with CDV virulent strains (Snyder-Hill). All dogs were protected against this challenge with only a few clinical signs being displayed [103]. Therefore, the idea of developing a cross-species infection in humans still poses a threat, since a punctual mutation in H protein in vitro allows CDV to infect cells using the human SLAM receptor [57].

## Conclusions

This review summarizes the most important aspects of CDV tropism and pathogenesis from a molecular perspective, comprising not only the viral protein interactions with the host cell receptors but also the influence of host factors on CDV virulence and the development of different pathologies from neurological to gastric clinical signs. The use of diverse receptors delimits CDV’s cellular tropism since lymphoid, epithelium, and CNS cells have different receptors that are implicated in the CDV infection at different stages, suggesting that the pathogenesis derived from a particular tropism is receptor-dependent.

CDV pathogenesis is quite diverse and dynamic due to its wide tropism spectrum. Understanding the mechanism by which the CDV generates its virulence specifically in dogs, as molecular interactions between host cell receptors and viral proteins, helps in clarifying the tropism and pathogenesis of CDV more accurately and understanding the failure of vaccinations in some cases. Undoubtedly, a lack of information about CDV’s molecular interactions limits the analysis of its multi-tropism. However, a valuable amount of data can be deduced using a MeV infection. Thus, the molecular process of a CDV infection cycle is essentially understood based on the involved MeV mechanisms. The reverse genetics technologies have played an important role in the construction of this understanding, specifically through the study of the CDV H protein that allows one to elucidate certain responses from a wide range of CDV strains, since the interaction between CDV and its cellular receptors depends on the H protein. This fact becomes highly relevant because the first step in all CDV infections involves this viral protein. Thus, understanding the incidence of modifications in the primary structure of the H protein in CDV variants becomes a necessity.

CDV induces multiple pathogenic effects due to the different interactions between the viral particle and the host. Its interplay with the immune system and subsequently transient immunosuppression is considered crucial in the development of different clinical signs of CDV infection. This immunosuppression has been considered the result of the interaction between viral proteins, since their modifications inhibit the immunosuppression.

Morbillivirus, as CDV, are distributed among carnivores. Considering the proximity of humans to domesticated animals such as dogs, the above fact represents the need for constant treatment, considering that infections in non-human primates has already been demonstrated. Additionally, viral replication in human cell lines using human nectin-4 as the entry receptor allows one to wonder whether the CDV can initiate a cross-species event in humans because of virus adaption.

Hence, many computational studies and directed mutagenesis in silico*,* as preliminary tools, have proved that in vitro and in vivo experiments are necessary to establish a better understanding of actual vaccination problems, interspecies cross-transmission, and diverse pathological signs related to CDV, not only to develop new alternative therapeutic approaches and treat the symptoms shown by domesticated dogs but most importantly to avoid the interspecies cross-transmission of CDV to humans.

## Additional file

Additional file 1: **Table S1.** GenBank accession numbers of all isolates used to construct Fig. 2. (XLSX 11 kb)

## Abbreviations

CDV: Canine Distemper Virus
CNS: Central nervous system
DL: Demyelinating leukoencephalitis
dTom: Red fluorescent protein
EGFP: Enhanced green fluorescent protein
ESCRT: Endosomal Sorting Complex Required for Transport
MeV: Measles Virus
ORF: Open reading frame
PAMPs: Pathogen-associated molecular patterns
PBMCs: Peripheral blood mononuclear cells
PRR: Pattern-recognition receptors
RNP: Ribonucleoprotein complex
SLAM: Signaling Lymphocyte Activation Molecule
UTRs: Intergenic untranslated regions
